# High-throughput DNA metabarcoding for determining the gut microbiome of captive critically endangered Malayan tiger (*Pantheratigrisjacksoni*) during fasting

**DOI:** 10.3897/BDJ.11.e104757

**Published:** 2023-09-05

**Authors:** Mohamad Khairulmunir, Millawati Gani, Kayal Vizi Karuppannan, Abd Rahman Mohd-Ridwan, Badrul Munir Md-Zain

**Affiliations:** 1 Department of Biological Sciences and Biotechnology, Faculty of Science and Technology, Universiti Kebangsaan Malaysia, 43600 Bangi, Selangor, Malaysia Department of Biological Sciences and Biotechnology, Faculty of Science and Technology, Universiti Kebangsaan Malaysia 43600 Bangi, Selangor Malaysia; 2 Department of Wildlife and National Parks (PERHILITAN), KM 10 Jalan Cheras, Kuala Lumpur, Malaysia Department of Wildlife and National Parks (PERHILITAN), KM 10 Jalan Cheras Kuala Lumpur Malaysia; 3 Centre for Pre-University Studies, Universiti Malaysia Sarawak, 94300, Kota Samarahan, Malaysia Centre for Pre-University Studies, Universiti Malaysia Sarawak, 94300 Kota Samarahan Malaysia

**Keywords:** *Pantheratigrisjacksoni*, carnivora, next generation sequencing, 16S rRNA, metabarcode

## Abstract

The Malayan tiger (*Pantheratigrisjacksoni*) is a critically endangered species native to the Malaysian Peninsula. To imitate wild conditions where tigers do not hunt every day, numerous wildlife sanctuaries do not feed their tigers daily. However, the effects of fasting on the gut microbiota of captive Malayan tigers remains unknown. This study aimed to characterise the gut microbiota of captive Malayan tigers by comparing their microbial communities during fasting versus normal feeding conditions. This study was conducted at the Melaka Zoo, Malaysian Peninsula and involved Malayan tigers fasted every Monday. In total, ten faecal samples of Malayan tiger, two of Bengal tiger (outgroup) and four of lion (outgroup) were collected and analysed for metabarcoding targeting the 16S rRNA V3–V4 region. In total, we determined 14 phyla, 87 families, 167 genera and 53 species of gut microbiome across Malayan tiger samples. The potentially harmful bacterial genera found in this study included *Fusobacterium*, *Bacteroides*, *Clostridium* sensu stricto 1, *Solobacterium*, *Echerichiashigella*, *Ignatzschineria* and *Negativibacillus*. The microbiome in the fasting phase had a higher composition and was more diverse than in the feeding phase. The present findings indicate a balanced ratio in the dominant phyla, reflecting a resetting of the imbalanced gut microbiota due to fasting. These findings can help authorities in how to best maintain and improve the husbandry and health of Malayan tigers in captivity and be used for monitoring in ex-situ veterinary care unit.

## Introduction

Tigers (*Pantheratigris*) are the largest felid species and a widely recognised symbol of worldwide wildlife conservation. Six tiger subspecies exist in the wild, including the Malayan tiger (*Pantheratigrisjacksoni*) (Luo et al. 2004, O'Brien et al. 2005), a charismatic iconic flagship species native to the Malaysian Peninsula. The Malayan tiger is currently classified as critically endangered (Kawanishi 2015) and totally protected in Malaysia under the Second Schedule of the Wildlife Conservation Act of 2010. The 1st National Tiger Survey (1st NTS) 2016–2018 found a Malayan tiger population of < 200 individuals. The Malayan tiger is threatened by anthropogenic disturbances, such poaching, forest clearance for agriculture, commercial and illegal logging and human settlements (Ten et al. 2021).

In the Malaysian Peninsula, the Department of Wildlife and National Parks (PERHILITAN) Malaysian Peninsula is the main authority responsible for managing and conserving the Malayan tiger. For instance, a project started in 2015 by the PERHILITAN under the 10^th^ Malaysian National Tiger Conservation Action Plan NTCAP aimed to potentiate the conservation management of the Malayan tiger (PERHILITAN 2008). The National Wildlife Rescue Center (NWRC) and Malayan Tiger Conservation Center (MTCC) are two of the *ex-situ* tiger conservation centres. The NWRC in Sungkai Wildlife Reserve offered facilities for breeding and rescuing wildlife; meanwhile, the MTCC in Krau Wildlife Reserve is building facilities for rewilding Malayan tigers (Malaysian Palm Oil Industry 2020). In addition, zoological parks also contribute to the conservation of Malayan tigers as well as providing educational and tourism benefits (Boyd et al. 2014).

*Ex-situ* platforms are indispensable to conserve the biodiversity of large breeding groups of mammals, such as tigers and their prey species (Ten et al. 2021). Numerous efforts have been aimed at improving the husbandry and welfare of Malayan tigers in captivity, including their health development through veterinary care, health screening and diet regime. Zoos in the Malaysian Peninsula provide them with different types of meat, such as chicken, beef and lamb and/or even live prey animals for hunting enrichment. In addition, most food regimes applied by zoos involve either daily feeding or one day fasting per week. The latter aims to imitate the conditions of a wild habitat where tigers do not hunt every day (De Cuyper et al. 2019). For instance, Amur tigers do not have a consistent diet in the wild. They could eat a lot at one meal and then skip meals for a few days (Ning et al. 2020). According to "The Times of India", all animals in Delhi Zoo are maintained fasting once a week, which helps maintain their health, as it improves their digestive systems (Sinha 2003). Fasting produces physical responses that prevent degradation of body tissues (Castellini and Rea 1992). With fasting, the metabolic rate slows down, decreasing the amount of nutrients that must be metabolised to cover maintenance requirements. In humans, fasting promotes a lower, maintained metabolic rate, decreased protein and carbohydrate catabolism and increased lipid oxidation to satisfy their increasing energy needs (Wang and Wu 2022). These changes spare proteins while depleting lipid stores (Goodman et al. 1984).

Concerning the effects of the fasting and non-fasting day on the condition of big cats in captivity, especially to their health, one of the studies that can be done is the study of the gut microbiota. The composition of gut microbiota is closely associated with the metabolism, health, nutritional status and immune system of the host (Iebba et al. 2012). It is interesting to determine the effect of fasting in contrast to daily eating in the gut health of big cats in captivity, particularly with respect to their gut microbiota content. Previous studies showed that the gut microbiota composition changes according to changes in host diet and environment (Candela et al. 2012, McKenzie et al. 2017, Allan et al. 2018). Animals in captivity go through various changes, such as dietary modifications or restrictions; in addition, veterinary treatments probably impact their microbiota as well (Hyde et al. 2016). Fasting or a temporary reduction in food intake has been shown to affect the gut microbiota in tadpoles (Kohl et al. 2014, Vences et al. 2016). The gut microbiota also improves survival during fasting periods (Alrubaye and Kohl 2021).

DNA metabarcoding is an advanced technology that uses high-throughput sequencing of a specific DNA marker to identify multiple species from a mixed sample to create barcodes from every organism in the sample (Darling et al. 2017, Liu 2020). Most animal metabarcoding markers used for this method are found in mitochondrial ribosomal RNA (rRNA) genes, such as the 12S rRNA and 16S rRNA genes (Chan et al. 2020, Chan et al. 2022, Daugaliyeva et al. 2022, Zhao et al. 2023). Metabarcoding using 16S rRNA markers has been used to map the microbiome of Asian flying fox, wild and captive chimpanzees, giant pandas and marmosets (Uenishi et al. 2007, Guo et al. 2019, Malukiewicz et al. 2022, Mohd-Yusof et al. 2022), for diet determination of Asian elephants using ribulose-1,5-bisphosphate carboxylase/oxygenase gene (rbcL) and transfer RNA-Leu (trnL) markers (Abdullah-Fauzi et al. 2022, Mohd-Radzi et al. 2022), diet determination in salamander using Cytochrome c oxidase I markers (Marques et al. 2022) and disease identification (Kocher et al. 2016, Batovska et al. 2017, Wilkinson et al. 2021). In the gut microbiota, DNA metabarcoding is used to identify host-associated microbial communities crucial to the health of hosts; for instance, studies of microbial communities in faecal samples are a non-invasive, precise, as well as time and cost-effective approach (Ando et al. 2020). Gut microbiota composition is significantly influenced by external stimuli, such as host factors, delivery pattern, age, diet and antibiotics (Hasan and Yang 2019). The immune system can be strengthened, indigestible substances can be metabolised, sickness can be prevented and nutrients can be produced by a healthy gut flora (Moeller et al. 2016).

Metabarcoding procedures are used most frequently for microbial community analysis by analysing the sequence of the PCR-amplified 16S rRNA marker (Fadeev et al. 2021). According to previous studies, a lion's feeding schedule, more closely resembling natural patterns, increased digestibility, lowered body weight and reduced pacing on fast days without affecting behaviour (Altman et al. 2005). The Melaka Zoo in the Malaysian Peninsula is one institution where the feeding regime for big cats includes one day fasting per week and provides easy access to collect faecal samples. Furthermore, fasting one day in a week is essential for adult tigers that consume around 10 kg meat daily (Shukla et al. 2014). In this study, we aimed to provide a better understanding of the gut microbiota of captive Malayan tigers and to compare the microbial community during fasting versus daily feeding conditions to contribute to the development of improved captive animal management strategies to improve the husbandry of big cats.

## Data Resources

**Sample Collection**

A total of 16 fresh faecal samples were collected and grouped, based on two conditions, fasting day (n = 8) and normal day (feeding day, n = 8). The total number of samples collected included 10 faecal samples of Malayan tiger (*Pantheratigrisjacksoni*), two of Bengal tiger (*Pantheratigristigris*) and four of lion (*Pantheraleo*). Bengal tiger and lion samples were used as an outgroup in this study. Faecal samples were collected using sterile gloves and spoon and kept in vials filled with absolute ethanol for preservation purposes and to avoid DNA degradation. All faecal samples were then stored at −20°C until DNA extraction.

**Study Area**

**Figure 1.**
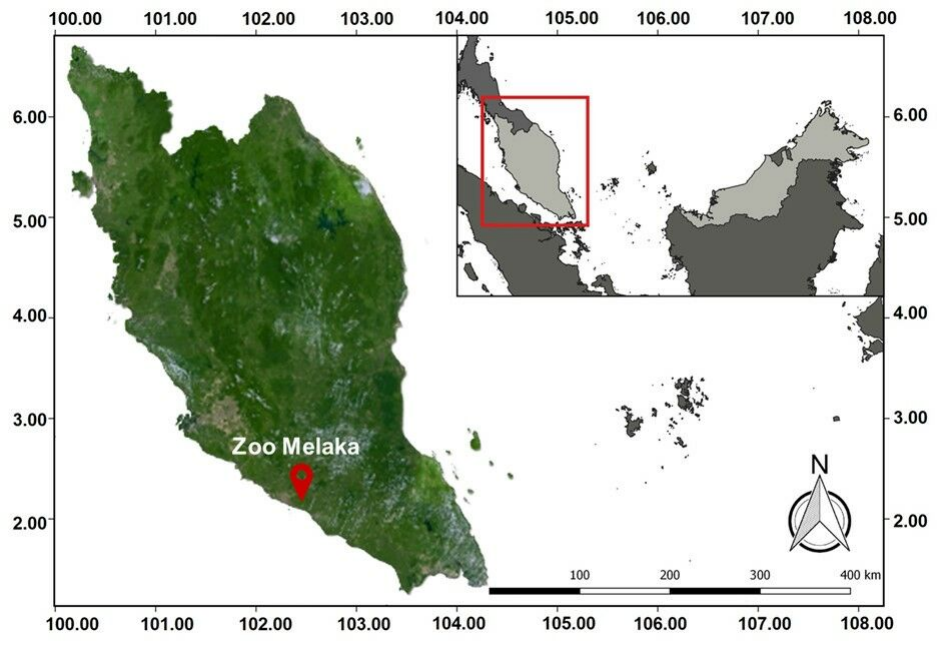
Zoo Melaka Map.

This study was conducted at the Zoo Melaka located in Ayer Keroh, Melaka, Malaysian Peninsula, at coordinates 2.2766°N, 102.2987°E (Fig. 1). This area is involved in preservation and conservation activities for Malayan tigers and other wildlife. Zoo Melaka has implemented a weekly fasting day (every Monday) for their captive big cats. Big cats have their last meal before fasting on Sunday evening; their next feeding is scheduled for Tuesday morning.

## Material and methods

### DNA Extraction

A total of 400 mg of each faecal sample were used to extract bacterial genomic DNA using innuPREP Stool DNA Kit (Analytik Jena, Germany), following the manufacturer’s protocol (Osman et al. 2020, Abdul-Latiff and Md-Zain 2021). The presence of purified DNAs was first visualised on a 1% Tris-acetate-EDTA (TAE) agarose gel electrophoresis. The concentration quality of DNA was measured using a spectrophotometer (Implen NanoPhotometer® N60/N50), while fluorometric quantification was performed using an iQuant™ Broad Range dsDNA Quantification Kit.

### 16s rRNA Gene Amplification and Sequencing

The 16 purified gDNAs were submitted to a service provider, Apical Scientific Sdn. Bhd. for 16S rRNA gene amplification and amplicon sequencing. The 16S rRNA gene was amplified by targeting the V3–V4 region using locus-specific bacterial 16S rRNA V3–V4 (Caporaso et al. 2012) primer sets with overhang adapters Forward: 5’-TCGTCGGCAGCGTCAGATGTGTATAAGAGACAG-CCTACGGGNGGCWGCAG-3’ and Reverse: 5’-GTCTCGTGGGCTCGGAGATGTGTATAAGAGACAG-GACTACHVGGGTATCTAATCC-3’. All PCR reactions were carried out using KOD -Multi & Epi-® (Toyobo), following manufacturer’s instructions. Dual indices were attached to the amplicons PCR using Illumina Nextera XT Index Kit v.2, following manufacturer’s instructions. The quality of the libraries was measured using an Agilent Bioanalyzer 2100 System by Agilent DNA 1000 Kit and fluorometric quantification was performed using a Helixyte GreenTM Quantifying Reagent. Finally, the libraries were normalised and pooled, followed by sequencing with the MiSeq platform using 300 paired-end sequencing, according to manufacturer’s instructions (Illumina Inc., San Diego, CA, USA).

### Statistical Analysis

All next-generation sequencing (NGS) data were uploaded into the National Center of Biotechnology Information (NCBI), under the Sequence Read Archive (SRA) Bioproject accession number PRJNA896752 and the individual Biosample accession numbers corresponding to each sample indicated in Table 1. Quality assessment of raw reads was performed using fastqc; thereafter, primers and adaptors were removed using Cutadapt 3.5 (Martin 2011, Osman et al. 2022). Then, the paired-read end was processed and merged using DADA2 V.1.18 (Callahan et al. 2016). Later, chimera screening and taxonomy ASV assignment were performed using DADA2 V.1.18 against the SILVA nr database V.138.1.

All statistical analysis was performed in Rstudio version 2022.07.2. To validate the sufficient depth of sequencing, we generated the rarefaction curves of the ASV number. Two series of alpha diversity indices including Shannon and Chao1 were calculated and analysed using the number of ASVs in Rstudio packages phyloseq (McMurdie and Holmes 2013) and ggplot2 (Wickham 2016). The Wilcoxon rank-sum test was performed to identify differences in microbiota composition between fasting and normal feeding phases. For beta diversity, principal coordination analysis (PCoA) was applied, based on weighted unifrac and the Bray-Curtis distance method. Differences in beta diversity were tested using a Permutational Multivariate Analysis of Variance (PERMANOVA) in vegan packages where p-values less than 0.05 are considered significant (Oksanen et al. 2016).

## Results

**Figure 2.**
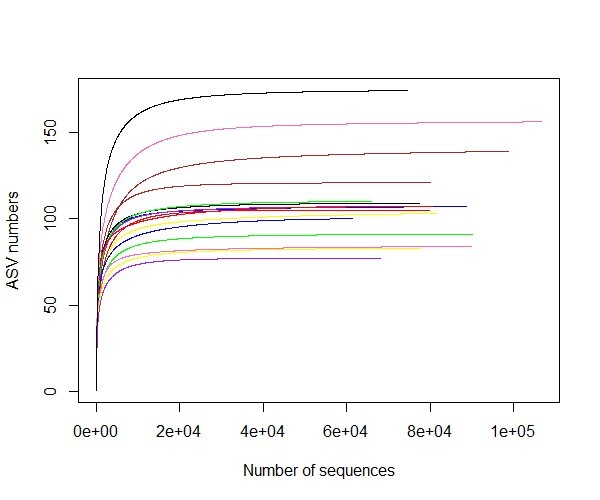
Rarefaction curve of the 16S rRNA gene sequence of the genus *Panthera* in Zoo Melaka calculated for operational taxonomic units at 97% similarity.

NGS produced 2,100,214 raw reads generated at the 97% similarity cut-off from 10 samples of *Pantheratigrisjacksoni* from Zoo Melaka, ranging from 112,622 to 171,950. NGS also produced 278,313 reads from two samples of *Pantheratigristigris* and 529,143 reads from four samples of *Pantheraleo*. A total of 1,299,189 non-chimeric sequence reads were obtained after filtering, denoising and merging. The highest non-chimeric sequences (106,804) were shown by P-NINI, followed by N-KINGKING (98,910) and Elsa (90,208) during the normal feeding phase. Fig. 2 shows the rarefaction curves between the number of sequences and ASVs, plotted with the help of the 16S rRNA gene metabarcoding database. The rarefaction curves produce a curve pattern indicating sufficient sampling depth (Fig. 2). The number of ASVs generated from the 16 samples was 747. The Shannon–Wiener index (H’) showed that, during fasting, Simba has the highest diversity (H’ = 3.6438 with 109 ASVs) and KingKing the lowest (H’ = 2.4274 with 136 ASVs) (Table 2). The Chao1 index showed that P-KINGKING had the highest diversity with a Chao1 value of 137.5, while P-NINI had the lowest diversity with a Chao1 value of 83.0.

**Microbial richness and composition of the microbiome**

**Figure 3.**
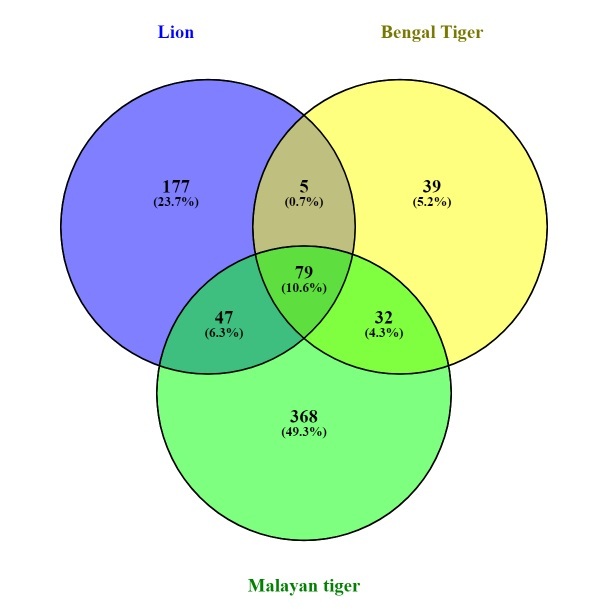
Venn diagram of ASVs detected in the gut microbiota of the Malayan tiger and outgroup.

**Figure 4.**
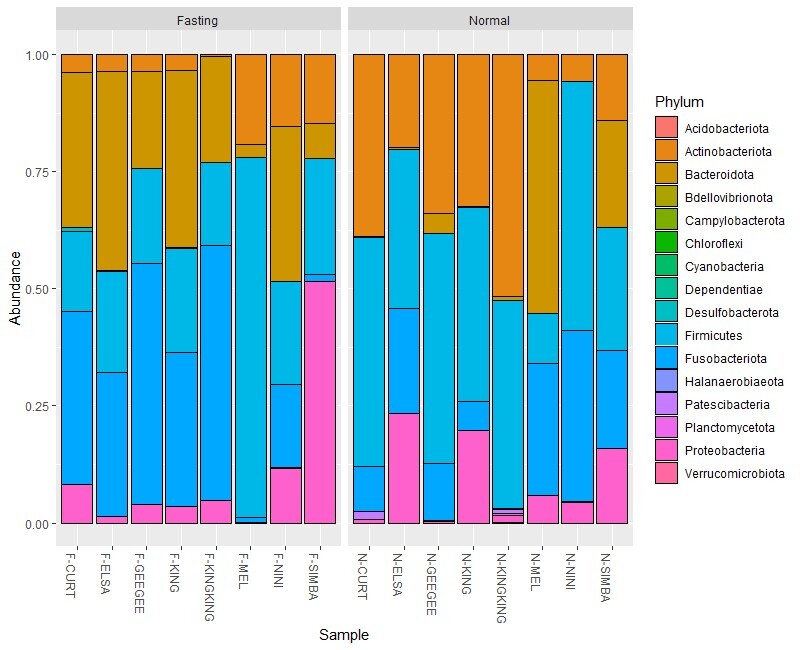
Relative abundance of gut microbiota ASVs (%) at the phylum level in the genus *Panthera* in Zoo Melaka.

**Figure 5.**
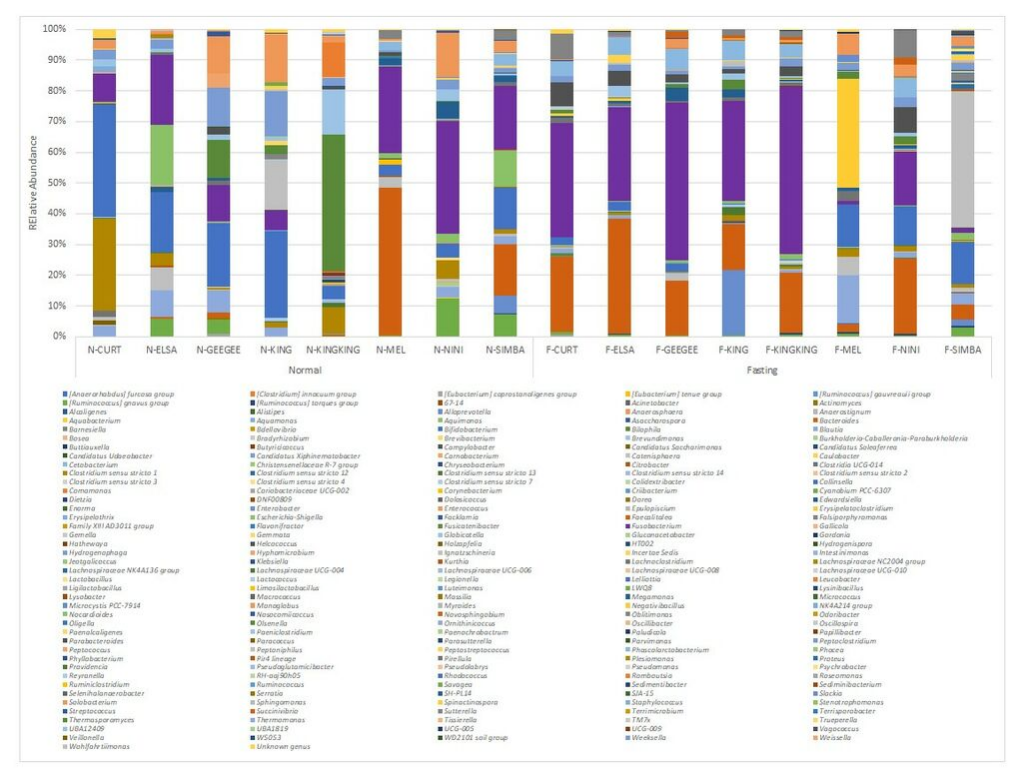
Relative abundance of gut microbiota ASVs (%) at the genus level in the genus *Panthera* in Zoo Melaka.

The ASVs were assigned to 14 phyla, 23 classes, 55 orders, 87 families, 167 genera and 53 species from 526 amplicon sequence variants across 10 Malayan tiger faecal samples. This study acquired 526 ASVs for Malayan tigers, 308 ASVs for lions and 125 ASVs for Bengal tigers. About 368 ASVs are unique to Malayan tigers, 177 ASVs for lions and 39 ASVs for Bengal tigers and 79 ASVs were common across all samples (Fig. 3). Furthermore, the ASVs were assigned to 16 phyla, 25 classes, 56 orders, 96 families, 201 genera and 72 species across all *Panthera* samples. Fusobacteriaceae is the highest family recorded in the fasting and normal feeding phases, 28% and 17%, respectively. The largest class identified during the fasting phase was Fusobacteria (28%), whereas that during the normal feeding phase was Clostridia (27%). Fusobacteriales (28%) was the most prevalent order in the fasting phase, whereas Coriobacterialis (25%) was the most prevalent in the normal feeding phase. The highest phylum identified during the normal feeding phase was Firmicutes (38%) and that during the fasting phase was Fusobacteriota (28%) (Fig. 4). Fusobacterium was the most prevalent genus observed in both the normal feeding and fasting phases (17% and 28%, respectively; Fig. 5). Table 3 lists the top 10 microbial genera identified and their abundances, from highest to lowest. Amongst the top 10 microbial genera detected in this study, seven could be classified as pathogenic, including *Fusobacterium*, *Bacteroides*, *Clostridium* sensu stricto 1, *Solobacterium*, *Echerichiashigella*, *Ignatzschineria* and *Negativibacillus*. Pathogenic bacteria could be found in both fasting and normal feeding phases. *Clostridium* sensu stricto 1, *Solobacterium* and *Ruminococcusgnavus* were detected during fasting, while *Ignatzschineria*, *Negativibacillus* and *Parabacteroides* were discovered during normal feeding.

**Figure 6.**
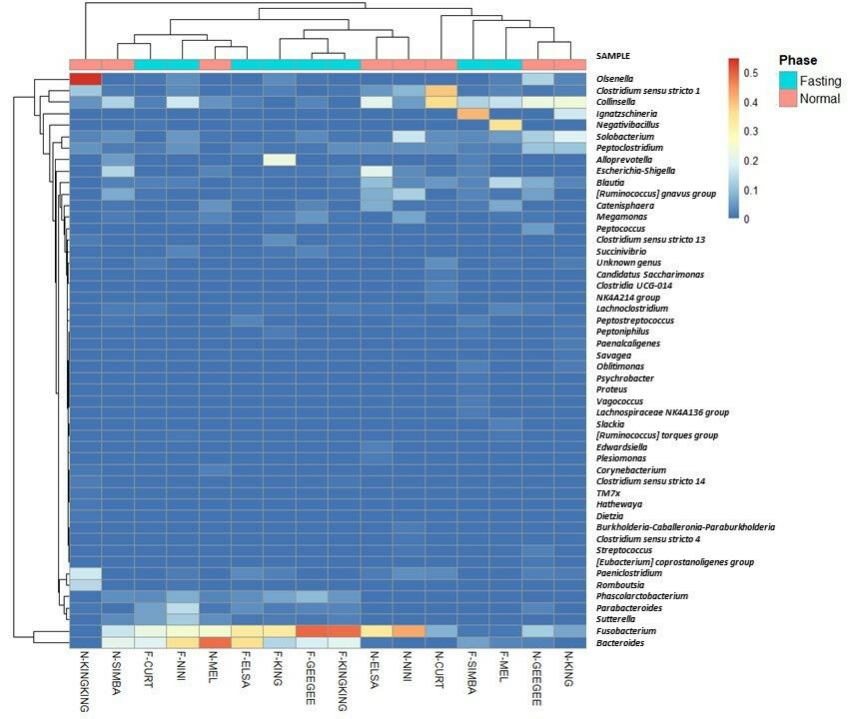
Heatmap including a dendrogram at the genus level using a gradient heatmap (over 1% of the microbiota).

Fig. 6 shows a heatmap of the 50 most predominant genera used in hierarchical clustering to evaluate the relationships amongst the 10 *P.t.jacksoni* individuals, two *P.t.tigris* individuals and four *P.leo* individuals using weighted pair clustering, based on Bray-Curtis measurements. Darker colours indicate lower abundance, whereas red indicates higher abundance.

**Figure 7.**
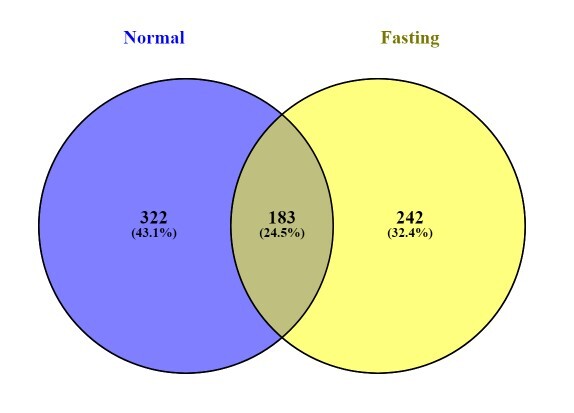
Venn diagram of ASVs detected in the gut microbiota of the genus *Panthera* in Zoo Melaka during normal feeding and fasting phases.

**Figure 8.**
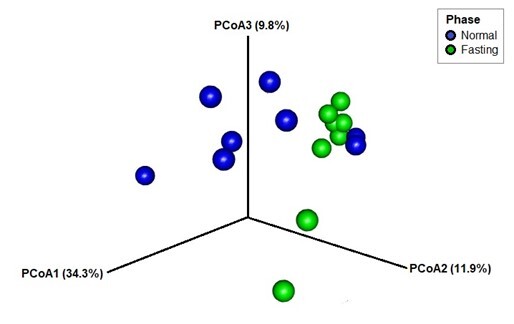
Three-dimensional plot of principal coordinate analysis (PCoA), based on weighted Unifrac (PCoA1 is variable at 34.3%, PCoA2 is variable at 11.9% and PCoA3 is variable at 9.8%).

Based on the Venn diagram in Fig. 7, 183 (24.5%) bacterial ASVs were shared amongst the two phases. The normal feeding phase had 322 ASVs (43.1%) with unique sequences, more than in the fasting phase (242 ASVs [32.4 %]). Regarding the beta diversity, PCoA UniFrac weighted distances showed significant differences between fasting and feeding phases (p-value < 0.05). Most samples were clustered together within the feeding phase group. Similarly, seven out of the eight plots for the fasting phase were grouped together, with half of the plots for the normal feeding phase (Fig. 8).

## Discussion

In this study, we determined 14 phyla, 87 families, 167 genera and 53 species of gut microbiome across Malayan tiger samples. As comparison with outgroups, we further identified 16 phyla, 96 families and 201 genera in the microbiome of the genus *Panthera* in Zoo Melaka. Firmicutes were the most prevalent bacterial phylum in both normal feeding and fasting phases, with 38% and 28%, respectively, demonstrating the stable nature of this phyla in the gut of the genus *Panthera*. Overall, ASVs from the phyla Firmicutes, Actinobacteriota, Fusobacteriota and Bacteroidota dominated the gut microbiota of the genus *Panthera* in both phases of the investigation. Previous studies have reported similar bacterial genera associated with the gut microbiota of the genus *Panthera*, although some previously-reported genera were not isolated in the present study (Hua et al. 2020, Mittal et al. 2020, Chen et al. 2022). The findings of this study are consistent with earlier research on carnivores, particularly the genus *Panthera* (Ning et al. 2020, Jiang et al. 2020, Sun et al. 2021). From the 201 genera isolated from the faecal matter of the genus *Panthera* in Zoo Melaka, some contained non-pathogenic species. There is a high prevalence of potentially harmful bacteria including *Fusobacterium*, *Bacteroides*, *Clostridium* sensu stricto 1, *Solobacterium*, *Echerichiashigella*, *Ignatzschineria* and *Negativibacillus*. These same bacterial phyla have also been observed in the intestinal microbiotas of other mammals, such as horse, chickens, goat and cows (Costa et al. 2012, Tong et al. 2022, Mahayri et al. 2022). This indicates that these bacteria are exceptionally well-suited to the mammalian gut environment. However, the considerable prevalence noted in this study should raise concern. As a result, these bacteria might be seen as opportunistic pathogens, given that they do not usually cause disease in healthy individuals, but can turn pathogenic under specific conditions (Davis 1996, Fouhse et al. 2016).

### Comparison of diversity and abundance of the gut microbiota in the genus Panthera between normal feeding and fasting

We found higher gut microbiota alpha diversity in the fasting phase compared to the normal feeding phase. Interestingly, the gut microbiota in captive *Panthera* shows higher community richness during fasting than during normal feeding phases. In terms of beta diversity, according to PCoA analysis, based on the Bray-Curtis distance, although the samples were separated by phases, no clusters by species were observed. This indicated significant differences in the gut microbiota community between the two phases. Jiang et al. (2020), who did a study on Bengal tigers, Amur tigers and south China tigers using meat diet, mixed diet and milk-fed diet, also found significant differences in the diet group, even though the diet variable selection is different from our study. Thus, any diet type or diet regime might affect the differences of the gut microbiota community. In normal feeding, the dominant phyla are Firmicutes followed by Actinobacteria, Fusobacteria and Bacteroidetes. Meanwhile, the fasting phase showed a balance in the abundance of gut microbiota which are Fusobacteria, Firmicutes and Bacteroidetes.

There was a high abundance of *Fusobacterium* in both normal feeding and fasting phases (28% and 17%, respectively.). Fusobacteria populate the mucous membranes of humans and animals and are thought to be commensals of the upper respiratory and gastrointestinal tracts (Ahmed et al. 2020). They develop long filamentous rods with pointed ends, which are sometimes described as fusiform or spindle-shaped (Allaker 2012). *Fusobacterium* species are all non-fermentative or rather mildly fermentative. All the studied *Fusobacterium* species can use glucose, which is integrated into cellular components (Hofstad 2006). The abundance of Fusobacteria is higher in the normal feeding phase as compared to the fasting phase. This could be due to the lower amount of glucose being produced during the fasting phase. During short-term fasting, the liver primarily generates and releases glucose via glycogenolysis. Glycogen is reduced during extended fasting and hepatocytes manufacture glucose by gluconeogenesis using lactate, pyruvate, glycerol and amino acids (Rui 2014).

During normal feeding, the abundance of the phylum Firmicutes in the genus *Panthera* was significantly greater than that of Bacteroidetes. The food intake during the feeding day may have decreased the abundance of Bacteroidetes. The increased abundance of Firmicutes in the normal feeding phase might be related to the protein intake during feeding. This finding is similar to that of Hua et al. (2020), which showed a relatively higher proportion of Firmicutes and lower proportion of Bacteroidetes in captive north China leopards than in wild leopards. This could be because captive animals are fed a set amount of food every day, while wild animals hunt on their own. In addition, the ratio of Firmicutes to Bacteroidetes is often linked to a rise in low-grade inflammation and a greater amount of energy extracted from food. This can increase blood sugar and fat levels, which can damage blood vessels and cause inflammation. Being overweight or obese is linked to both factors (Stojanov et al. 2020, Magne et al. 2020, Houtman et al. 2022). Interestingly, during fasting, there was a balanced ratio between Firmicutes and Bacteroidetes. This showed the exercise of fasting day in big cats helps to reset the abundance of certain bacteria for shaping the gut microbiota to benefit health and suggest fasting in the big cat’s diet regime is recommended in captivity.

Bacteria of the *Clostridium* species are generally acknowledged as harmful and indicative of a not-so-healthy microbiota (Stackebrandt et al. 1999, Rajilić-Stojanović and de Vos 2014). Thus, this genus could be a potentially pathogenic bacterium, especially in Malayan tigers. This study would recommend that Zoo Melaka maintains its current *Panthera* food regime by fasting their big cat at least one day per week. The fasting phase in this study may serve as a resetting of the imbalance of gut microbiome composition. The fasting phase implemented at Zoo Melaka is characterised as short-term and it could potentially result in a temporary decrease the prevalence of certain bacteria and restoration of a balanced gut microbiome diversity in *Panthera*. Hence, this study also suggests that future studies should explore the effects of longer-term fasting or restricted time feeding regime on the gut microbiome diversity of the *Panthera* in captivity. This would provide insights into the potential impacts of extended fasting periods and another food regime of captive *Panthera*. The genus *Solobacterium* was found in the gut of genus *Panthera* during the normal feeding phase. However, *Solobacteriummoorei* is the only member of *Solobacterium* (Kageyama and Benno 2000). *Solobacteriummoorei* is a Gram-positive, non-spore-forming, obligate anaerobic bacillus from human faeces. Previous faecal microbiota metagenomic analysis revealed various anaerobes including *S.moorei* that could cause colorectal cancer (Yu et al. 2015). This shows that both phases comprised of their own distinct potential pathogenic bacteria. However, these pathogenic bacteria do not cause major diseases to the big cats at Zoo Melaka. This could be due to the Zoo's excellent management on captive big cats including its feeding diet plan.

## Conclusions

Our findings indicate that fasting increased gut microbial richness in *Panthera* in captivity. Particularly, we observed a balanced ratio in the dominant phylum, which may reflect the effect of fasting in resetting imbalances in the gut microbiota. Seven of the top 10 microbial genera identified in this study can be classified as pathogenic. However, this does not impact the health of the big cats in Zoo Melaka. A fasting day is essential to captive big cats to improve their gut health. The findings revealed a balanced ratio in the dominant phylum, which may reflect the effect of fasting activities in resetting the imbalance of microbiota in the gut. Overall, we could identify potential pathogenic bacteria unique to both phases. Future studies should increase the number of fasting days to better explain the differences between the phases. In the future, the sample size should include samples from all captive areas in Malaysia and an increase in the frequency of sample collection may yield better outcomes. Enrichment activities should be considered for future studies to evaluate if there is a benefit in lowering harmful bacteria in this carnivore's gut microbiota. The present results can provide information for conservation efforts and improve the knowledge on the impact of man-made environments on animal health and disease control.

## Figures and Tables

**Table 1.** List of samples used for microbiota analysis taken at fasting (P) and normal feeding (N) phase.

#	**Sample Name**	**Species**	**Common name**	**Accession Number**
1	P-Curt	*P.t.jacksoni*	Malayan Tiger	SRR22137820
2	P-GeeGee	*P.t.jacksoni*	Malayan Tiger	SRR22137905
3	P-KingKing	*P.t.jacksoni*	Malayan Tiger	SRR22137933
4	P-Mel	*P.t.jacksoni*	Malayan Tiger	SRR22138007
5	P-Nini	*P.t.jacksoni*	Malayan Tiger	SRR22138012
6	P-Elsa	*P.t.tigris*	White Bengal Tiger	SRR22138194
7	P-King	*P.leo*	White African Lion	SRR22138207
8	P-Simba	*P.leo*	White African Lion	SRR22138208
9	N-Curt	*P.t.jacksoni*	Malayan Tiger	SRR23269158
10	N-GeeGee	*P.t.jacksoni*	Malayan Tiger	SRR23269159
11	N-KingKing	*P.t.jacksoni*	Malayan Tiger	SRR23269394
12	N-Mel	*P.t.jacksoni*	Malayan Tiger	SRR23269395
13	N-Nini	*P.t.jacksoni*	Malayan Tiger	SRR23269492
14	N-Elsa	*P.t.tigris*	White Bengal Tiger	SRR23269493
15	N-King	*P.leo*	White African Lion	SRR23269558
16	N-Simba	*P.leo*	White African Lion	SRR23269583

**Table 2.** Number of observed amplicon sequence variants and alpha diversity indices for the bacterial DNA from all samples at fasting (P) and normal feeding (N) phase.

**Individual**	**Non-chimeric Sequence**	**ASVs**	**Shannon H**’	**Chao1**
P-CURT	61314	107	2.53	107.5
P-ELSA	65943	91	2.89	91.0
P-GEEGEE	77585	101	2.97	104.0
P-KING	80465	105	3.04	105.0
P-KINGKING	80023	136	2.43	137.5
P-MEL	68135	106	3.07	106.0
P-NINI	106804	83	3.08	83.0
P-SIMBA	74547	109	3.64	109.0
N-CURT	88859	100	3.46	100.6
N-ELSA	90208	109	3.38	109.0
N-GEEGEE	81777	81	3.09	81.0
N-KING	79941	106	3.51	106.8
N-KINGKING	98910	121	3.37	121.0
N-MEL	77206	77	2.63	77.0
N-NINI	89986	154	3.76	154.3
N-SIMBA	77486	174	3.36	174.5
**Total**	**1299189**	-	-	-

**Table 3.** Top 10 Microbial genera and abundance.

**Bacterial genus (Normal)**	**Abundance**	**Percentage (%)**	**Bacterial genus (Fasting)**	**Abundance**	**Percentage (%)**
*Fusobacterium*	114604	17	*Fusobacterium*	173254	28
*Collinsella*	111990	16	*Bacteroides*	112886	18
*Olsenella*	56890	8	*Collinsella*	39124	6
*Bacteroides*	52422	8	*Ignatzschineria*	33136	6
*Clostridium* sensu stricto 1	47879	7	*Phascolarctobacterium*	27908	5
*Solobacterium*	43719	6	*Negativibacillus*	25754	4
*Peptoclostridium*	34758	5	*Parabacteroides*	22907	4
*Escherichia*-*Shigella*	31827	5	*Sutterella*	19099	3
[*Ruminococcus*] *gnavus* group	26192	4	*Alloprevotella*	18676	3
*Paeniclostridium*	23969	3	*Blautia*	18118	2
